# Some Remarks upon Cases of Obstruction of the Bowels in Which Surgical Means Were Used

**Published:** 1871-06

**Authors:** A. Reeves Jackson

**Affiliations:** 785 Michigan avenue; Chicago, Ill.


### Chicago Medical Journal.

A MONTHLY RECORD OF

*Medicine, Surgery, and the Collateral Sciences.*

EDITED BY J. ADAMS ALLEN, M.D., LL.D.; AND WALTER HAY, M.D.

---

VOL. XXVIII.—JUNE, 1871.—No. 6.

---

### Original Communications.

ARTICLE I.—*Some Remarks upon Cases of Obstruction of the Bowels in which Surgical Means were used.* By A. REEVES JACKSON, M.D., Chicago, Ill.

(Continued from page 135.)

CASE III.—*Intussusception of the ileum—Peritonitis—Puncture of Bowel—Death.*

James W., aged 17 years, a house painter's apprentice, was awakened about midnight with violent spasmodic pains, a little below and to the right of the umbilicus. He arose and passed a natural stool, with temporary relief. The pain returned in a few minutes, and was accompanied with cramps in the thighs. He took two tablespoonsful of sulphate of magnesia, and had hot applications to the abdomen. The pain, which was described as being of a sharp, tearing character, and occasionally as though the bowel was constricted by a cord, continued, and a glass of hot, spiced whisky was taken two hours after the salts. This was almost immediately ejected by vomiting. During the next day the abdomen became somewhat distended, and nausea and vomiting were quite frequent, the ejected matter consisting of a yellowish fluid, having an unpleasant but not feculent odor.

He came under the care of the writer September 17th, 1868, two days after the commencement of the attack. At this time he was pale, and his expression somewhat contracted; pulse 120, small, quick and wiry; tongue dry, and covered with a yellowish-brown fur. His gums presented a bluish margin. The abdominal walls were moderately distended and resonant, except in the right iliac fossa, where a hard, somewhat cylindrical tumor was distinctly felt, its axis lying across the abdomen, and extending beyond the median line. The urine had been passed in normal quantity throughout the attack. The rectum was empty.

Here were spasm, constipation, abdominal pain, blueness of gums, and the fact that the patient had been engaged in an occupation that exposed him to the influence of lead poison. On the other hand, there was the existence of a suddenly formed tumor, which, when taken in connection with localized pain, obstinate constipation, vomiting, and the passage of blood, constitutes the most distinctive and reliable sign of intussusception. Although in this case there had been no appearance of blood, I regarded the symptoms sufficiently well marked to warrant a diagnosis of the last-named condition.

Hot fomentations and poultices were applied to the abdomen, and a large enema of thin gruel, administered through a long, flexible tube, passed carefully into the colon. This was returned, in about half an hour, unchanged. A full dose of sulphate of morphia, in combination with two grains of calomel, was given, with orders for its repetition every four hours.

18th. All the symptoms were worse. The abdominal pain was intense and constant; the stomach rejected everything; substances vomited were now plainly stercoraceous, and contained blood. The patient's countenance was pale, haggard, and anxious; skin cool and dry; pulse 130, small, thready and weak; respirations 30, but much quickened with slight exertion; tongue dry and cracked. He was lying on his back, with the legs drawn up. The abdomen was enormously distended, and resonant in every part except the most dependent, while the integument covering it was tense and shining. Urine was passed in full quantity, without the patient rising. Several enemata of warm water, thin broths, etc., each containing laudanum, had been given, but all were quickly returned, some tinged with blood, while others were unchanged.

Believing that the greater part of the patient's sufferings arose from the excessive tympanites, I punctured the abdomen at a prominent point just below the umbilicus, by means of a small exploring trocar. This was followed by the escape, through the tube, of a large quantity of slightly fetid gas, with subsidence of the abdominal fulness, and great and immediate relief to the symptoms. The patient could now bear sufficient pressure upon the abdomen to enable me to feel the sausage-like tumor of the right side again, and I also discovered the presence of a considerable amount of effusion.

Hot fomentations, containing turpentine and opium, were now assiduously applied; mercurial ointment was rubbed into the skin, inside of the thighs; and suppositories, containing two grains of opium, were placed in the rectum every four hours.

After a time the patient fell into a disturbed sleep, which lasted two hours. On awaking he was exceedingly thirsty, and took a large draught of water. This produced vomiting, and a return of the pain. The abdominal distension also rapidly returned, and at ten o'clock in the evening—thirteen hours after the tapping—it was as great as before.

19th. This morning found the patient still worse. The abdomen was stretched to its utmost capacity. The superficial veins were turgid and prominent, and the skin glistening. The knees were drawn closely up to the chest. The surface was cool and moist; pulse 120, small and tense; respirations 30; urine somewhat scanty and highly colored. He is tormented with insatiable thirst, and drinks plentifully, everything being instantly rejected. The vomited matters are mostly clear, consisting of the water swallowed, together with a yellowish-brown substance, having a sour, unpleasant, feculent odor, and sometimes turbid.

The gas was again drawn off by means of the trocar, but the relief afforded was not nearly so great as before. During the day he steadily grew worse. The feculent vomiting became almost constant; delirium set in during the following night; he then became comatose, and died at 8 A. M., on the fifth day of the sickness.

The inspection was made eight hours after death. The abdomen was much enlarged from flatulent distension of the small intestine. The peritoneum was greatly injected, and its cavity contained nearly two pints of serum, and also collections of thin pus. Feeble adhesions, by means of recently-effused lymph, were observed between contiguous surfaces of the intestinal coils. On laying open the large intestine, a portion of the ileum, about six inches in length, was found invaginated in the cœcum and colon, and tightly constricted by the ileo-cœcal valve. Above the point of obstruction, the intestines were deeply congested, distended with gas, and contained also a large quantity of a yellow, pultaceous fœcal matter. Below it, the transverse colon was filled with gas, but the remainder of the bowel was empty and healthy.

*Remarks.*—No one whose attention has been directed to the literature of obstruction of the bowel, can fail to be struck with the fact that very many of the cases which have resulted fatally might, in all probability, have been saved by a timely surgical operation. In reading the accounts of the autopsies of such cases, how frequently do we find that the cause leading to the fatal result is stated to be a band of adhesion—a twisting of the bowel—slight intussusception—and other mechanical derangements, quite easily relieved by surgical interference, and yet almost certain to end in death without such interference. I am well aware of the fatality that has attended operations for the relief of internal strangulation, but cannot resist the conviction that in many of them the untoward result has been due, not to the operation itself, but to the delay in resorting to it. Perhaps there is more force in some other objections that have been urged against the use of surgical means in these cases. For example, the peritoneum is either already inflamed or in a state of intense congestion, and both conditions are likely to be aggravated by gastrotomy. But is the operation more certain to produce this result than a continuance of the strangulation? I think not; and if the obstruction is of such character as to be susceptible of relief by operation, surely the patient's chances for recovery are very much increased.

It has been urged, too, that the difficulties in the way of accurate diagnosis constitute an objection to gastrotomy. This objection had more weight a quarter of a century ago than at the present time; for, thanks to the labors of such men as Hilton, Brinton, Habershon and others, many cases, that in their diagnostic aspect were obscure at that time, are now comparatively clear. Nevertheless, it must be admitted that there are even yet few conditions that present more puzzling features than some of these cases of obstructed bowel. Still, even here, when the symptoms have been sudden in their onset, the pain acute, paroxysmal and limited to one spot, the constipation unyielding to laxatives, opium, the warm-bath, etc., and although we may be in doubt as to the exact nature of the obstruction, I would regard it as entirely proper to make an exploratory operation, guided by the site of pain. Operations of this kind, now so common in cases of obscure abdominal tumors, are attended with comparatively little danger, and are sufficient to decide the diagnosis, and determine the treatment. Indeed, if this whole question of surgical treatment were entertained at a much earlier period than it usually is, I do not doubt that the death-rate would be materially changed; for operative measures promise success in proportion to the promptness with which they are instituted. If delayed until peritonitis has taken place, and the involved structures are disorganized, we cannot, of course, reasonably expect success, and obloquy is attached to an operation which, if performed more timely, would be frequently useful.

We are told likewise that some of these cases, apparently of the most desperate character, recover. This is unquestionably true, and it is well known that an invaginated portion of intestine may become sphacelated at its extremities, detached from its place, and discharged from the body without necessarily destroying life. But while a knowledge of these facts may be sufficient to prevent us from regarding such cases as utterly hopeless, they are yet so rare and attended by so many risks of failure, that they do not encourage us to rely upon such unusual efforts of the *vis medicatrix* in the hour of need.

In many cases of obstruction, the excessive distension of the intestine appears to be not only the chief cause of distress, but likewise of inflammation, extravasation and paralysis of the organ. Here there should be no hesitation or delay in puncturing the bowel at a prominent point, and thus removing this source of pain and danger. Under such circumstances, I believe the operation to be almost harmless and unquestionably proper.

When insuperable obstruction arises from stricture of the rectum, whether high or low, colotomy should be performed. The colon may be opened in the loin, without injury to the peritoneum, thus relieving this proceeding from the objections that attach to those involving the integrity of that part. This operation is likewise demanded for irremovable tumors and cancer of the rectum. Although in these cases the operation has no power to cure the disease, it will, especially in the case of cancer, relieve the excruciating pain caused by the passage of fæces over the exquisitely sensitive bowel preventing sleep and destroying appetite, lessen greatly the amount of purulent and bloody discharge, and thus prolong life.

785 Michigan avenue.

